# *In vivo*-like Culture of Monophagous Animal Organ using Dietary Components

**DOI:** 10.26502/jbb.2642-91280070

**Published:** 2023-02-10

**Authors:** Norichika Ogata, Shogo Konishi, Takeshi Yokoyama

**Affiliations:** 1 Nihon BioData Corporation, 3-2-1 Sakado, Takatsu-ku, Kawasaki, Kanagawa 213-0012, Japan; 2 Laboratory of Sericultural Science, Faculty of Agriculture, Tokyo University of Agriculture and Technology, 3-5-8, Saiwai-cho, Fuchu, Tokyo, 183-8501, Japan

**Keywords:** Dedifferentiation, Monophagy, Primary Explant Culture, RNA-seq, Silkworm

## Abstract

Animals depend on other species to live, with monophagy being an extreme mode. Monophagous animals depend on their diet not only for nutritients but also for developmental and reproductive controls. Thus, dietary components may be useful in culturing tissues from monophagous animals. We hypothesized that a dedifferentiated tissue from the monophagous silkworm, *Bombyx mori*, would re-differentiate when cultured in a medium containing an extract of mulberry (*Morus alba*) leaves, the only food of *B. mori*. Over 40 fat-body transcriptomes were sequenced, and we concluded that it is possible to establish *in vivo*-like silkworm tissue cultures using their diet.

## 1. Introduction

Tissue culturing is performed to maintain isolated portions of multicellular organisms in an artificial milieu that is outside the individual organism over considerable periods of time. The technique is used not only to produce materials for bio-manufacturing [1] but also for studying plant and animal cell behaviors in environments free from systemic variations that might arise *in vivo* during both normal homeostasis and experimental stress. Conventionally, when cells are used to produce pharmaceutical proteins, a culture method that achieves a greater target productivity is required, but owing to the diversification of modalities, the cells themselves have become therapeutic agents. As a result, there is an increasing demand for culturing methods that provide mature cells [2]. How can we establish *in vivo*-like culture environments that provide mature cells? Tissues in the culture environment easily dedifferentiate and lose their mature characteristics [3,4]. To suppress dedifferentiation [5,6], methods of devising cell scaffolds [7,8] and the inclusion of gases in incubators [9], as well as methods using components derived from other species, have been proposed. Methods for suppressing dedifferentiation using components derived from a microorganism [10] and from tea [11] have been reported. Why are components derived from other species effective in suppressing cell culture environment-dependent dedifferentiation? In a relationship between a certain species and another species, the latter may be indispensable for the former’s establishment. Monophagous animals are extreme examples of such interspecies relationships. For example, Koalas eat only eucalyptus leaves, and silkworms eat only mulberry leaves [12]. When an animal is completely dependent on a forage plant for its diet, its cells alone are not sufficient to form a normal individual. In these relationships, the forage plant provides more than just nutrients, proteins, lipids, sugars, vitamins, minerals and water resources to the animal. For example, components derived from the leaves may promote the actions of animal organs or function like hormones. Therefore, we investigated the effects of adding diet-derived components to the medium of the primary cultured tissues of a monophagous animal, the silkworm *Bombyx mori*. In previous studies, the fat bodies of *B. mori*, a monophagous insect, were dedifferentiated in a primary culture environment [13]. A method to quantitatively evaluate the dedifferentiation of primary cultured tissues using a transcriptome has been established [13,14]. The previous work of the author-imposed significance of utilizing entropy in an excellent way in 2010, and its piercing insight lead to other fine articles [15,16]. Specifically, the information entropy of the transcriptome data can be used as an index of transcriptome diversity. The value decreases when a certain cell is in a differentiated state in the living body and expresses intensively a specific gene. The value increases when a certain cell tends to dedifferentiate in a culture environment and the expression of the specific gene is not promoted. A system that has lost control has increased entropy. The dedifferentiated cultured tissues may be re-differentiated by the addition of a high concentration of hydrophilic phenobarbital or hydrophobic permethrin [14]. The addition of these molecules, with their opposite affinities for water, result in similar responses. Thus, the tissue responses were not interpreted as being caused by a compound-specific bioactivity, but by the stimulation of a control system that exists *in vivo*. Thus, we aimed to identify the stimuli in this *in vivo* control system. In this monophagous insect, the fat body is located adjacent to the midgut, which is filled with chewed mulberry leaves. The fat body appears as a thin worm-like tissue wrapped around a mulberry-leaf sausage. Thus, we hypothesized that the mulberry leaf inside the midgut, or compounds from the leaves, contributes to the *in vivo* environment. Therefore, in this study, we investigated the effects of mulberry leaf-derived components on the transcriptomes of cultured silkworm fat bodies. Dedifferentiated ‘p50’ fat bodies were primary cultured in a medium containing a silkworm midgut extract and a medium containing a mulberry leaf extract, and changes in the information entropy of the transcriptomes were examined. In addition, fat bodies of ‘Sawa-J’, a broad-eating silkworms strain obtained by breeding, were used. The dedifferentiated ‘Sawa-J’ fat bodies were cultured in media containing silkworm midgut, mulberry leaf, or cabbage leaf extracts, and changes in the information entropy of the transcriptomes were examined. In addition, we examined the information entropies of the transcriptomes of the ‘Sawa-J’ fat bodies 12, 24, and 48 h after the start of culturing and compared them to those of *in vivo* ‘Sawa-J’ fat bodies. Silkworm fat bodies dedifferentiate 80 h after culturing [13,14], but it was unclear how many hours were sufficient for dedifferentiation.

## 2. Materials and Methods

### 2.1 Insects and Plants

The ‘p50’ and ‘Sawa-J’ strains of *B. mori* were reared on fresh leaves from the mulberry *Morus alba*. At 3 days after the 4^th^ ecdysis, 5^th^-instar larvae were harvested. Female insects were used. *Morus bombycis* was cultivated on a farm at the Tokyo University of Agriculture and Technology, Fuchu-shi, Tokyo, Japan. The cabbage *Brassica oleracea* var. *capitata* was obtained at a supermarket (Maruetsu, Kawasaki, Kanagawa, Japan).

### 2.2 Media Preparation

The MGM-450 insect medium (10% fetal bovine serum) was used in this study [17]. A 2-mL stock solution of silkworm midgut extract was prepared culturing isolated the midgut for 10 min. For culturing, the silkworm midgut extract’s stock solution was diluted 10-fold in the medium. The mulberry leaf extract’s stock solution was prepared by homogenizing 0.25 g of the leaves in 2 mL of medium using scissors (Dissecting Scissors Iris 11 cm D340-476; FRIGZ, Chiba, Japan). For culturing, the mulberry leaf extract’s stock solution was diluted 100-fold and 1,000-fold independently in the medium. The cabbage leaf extract’s stock solution was prepared by homogenizing 0.25 g of leaves in 2 mL of medium using scissors. For culturing, the cabbage leaf extract’s stock solution was used.

### 2.3 Culturing of ‘p50’ Fat Bodies in Mulberry Leaf Extract

The silkworm larvae were surface-sterilized by submersion in 70% ethyl alcohol, and the fat bodies were removed without injuring the digestive system. The intact fat bodies of four ‘p50’ larvae (Samples 1, 2, 3, and 4) were dissected, soaked in 0.35 mL of TRIzol LS (Invitrogen, CA, USA), and homogenized using a BioMasher II (Nippi, Tokyo, Japan) and a PowerMasher II (Nippi). Then, the tissues were maintained at −60°C until used. More than 100 fat body tissue samples with intact lobes (approx. 2 mm^3^) were excised from fat bodies dissected from 21 larvae. Tweezers (KFI K-23 No. 3 120 m/m 18-8 Stainless 230001; Kowa Pinset Industry, Tokyo, Japan) and scissors (FRIGZ) were used. These tissue samples were incubated in cell culture dishes (ø = 35 mm; AS ONE, Osaka, Japan) with 2 mL of MGM-450 insect medium and no gas change. The tissue samples were cultured without antibiotics for 80 h at 25°C. We examined the morphology of cultured fat body cells using a phase contrast microscope (ECLIPSE TS100; Nikon, Tokyo, Japan). The presence or absence of microbial contamination in the culture was monitored by microscopic inspection. Whole cultured tissues were mixed in a cell culture dish (ø = 100 mm; AS ONE) to eliminate the effects of inter-individual variation observed in primary cultures [18]. The original medium in each dish was replaced with an assay medium. In ‘p50’ controls, the original medium was replaced with fresh medium (Samples 5, 6, and 7). The primary cultured ‘p50’ tissues were incubated in two concentrations of mulberry leaf extract (mulberry 1/1,000: Samples 8, 9, and 10; and mulberry 1/100: Samples 11, 12, and 13). The primary cultured ‘p50’ tissues were incubated in medium containing silkworm midgut extract (Sample 14). After 10 h, the cultures were terminated by soaking the tissues in 0.35 mL of TRIzol LS, and tissues were then homogenized using a BioMasher II (Nippi) and a PowerMasher II (Nippi). Samples were maintained at −60°C until used.

### 2.4 Culturing of ‘Sawa-J’ Fat Bodies in Mulberry Leaf Extract

The larvae were surface-sterilized as described previously. The intact fat bodies of ‘Sawa-J’ larvae (Sample 15) were dissected and maintained at −60°C until used as described previously. More than 50 fat body tissue samples with intact lobes (approx. 2 mm^3^) were excised from fat bodies dissected from 12 larvae. These tissue samples were incubated 80 h and observed as described previously. Whole cultured tissues were mixed in a cell culture dish as described previously. The primary cultured ‘Sawa-J’ control tissues were incubated in fresh medium for 10 h (Samples 16, 17, and 18). The primary cultured ‘Sawa-J’ tissues were incubated in two concentrations of mulberry leaf extract for 10 h (mulberry 1/1,000: Samples 19, 20, and 21; and mulberry 1/100: Samples 22, 23, and 24). The primary cultured ‘Sawa-J’ fat bodies were incubated in a medium containing silkworm midgut extract for 10 h (Samples 25, 26, and 27) and in a medium containing cabbage leaf extract for 10 h (Samples 28, 29, and 30). The cultures were terminated as described previously.

### 2.5 Time Course of the ‘Sawa-J’ Tissue Cultures

The larvae were surface-sterilized as described previously. The intact fat bodies of three ‘Sawa-J’ larvae (0 h; Samples 31, 32, and 33) were dissected and maintained at −60°C until used as described previously. More than 30 fat body tissue samples with intact lobes were excised from fat bodies dissected from 9 larvae. These ‘Sawa-J’ tissue samples were incubated for 12 (Samples 34, 35, 36, 37, and 38), 24 (Samples 39, 40, and 41), and 48 (Samples 42, 43, and 44) h. The cultures were terminated as described previously.

### 2.6 RNA Extraction and Sequencing

Total RNA was extracted using TRIzol LS and a Direct-zol RNA Kit (Zymo Research, CA, USA) following the manufacturers’ instructions. The homogenates were incubated for 5 min at 25°C, and the samples were subjected to centrifugation at 12,000 ×g for 10 min at 5°C. The supernatants were then transferred to new tubes and incubated at 25°C for 5 min. An equal volume (approximately 0.35 mL) of ethanol was added to each sample homogenate and mixed thoroughly. The mixtures were then transferred into Zymo-Spin IIICG Columns in collection tubes and centrifuged. The flow-through was discarded, and columns were transferred into new collection tubes. Then, 400 μL Direct-zol RNA PreWash was added to each column, and the columns were centrifuged. The flow-through was discarded, and the prewash and centrifugation were repeated. Afterward, 700 μL RNA Wash Buffer was added to each column, and they were centrifuge for 1 min. The columns were carefully transferred into RNase-free tubes. To elute the RNA, 100 μL of DNase/RNase-free water was added directly to each column matrix, and the columns were centrifuged. The integrity of the rRNA in each sample was checked using an Agilent TapeStation 4200 (Agilent Technologies, CA, and USA). mRNA libraries were prepared for RNA sequencing using a TruSeq RNA Sample kit (Illumina, San Diego, CA, USA) in accordance with the manufacturer’s protocols. These libraries were sequenced using a NextSeq 500 sequencer (Illumina) in accordance with the manufacturer’s protocols. Short-read data were deposited in the Short Read Archive (project ID, DRA011291) of the DNA Data Bank of Japan. Short-read sequences were mapped to an annotated silkworm transcript sequence obtained from SilkDB [19] using hisat2 [20]. We did not use other full-length cDNA data set from National Agriculture and Food Research Organisation in 2011 (AK377185 - AK388575) since over 30% multi-hits were found.

### 2.7 Data Analyses

The Shannon entropies of the transcriptome data were calculated as described previously [14] using R (Supplemental Material 1). A t-test was performed on the null hypothesis, which stated that the mulberry leaf extract’s concentration did not affect the information entropy of the transcriptomes of the ‘p50’ fat bodies, using R [21] and Fitting Linear Models (lm) (Samples 5, 6, 7, 8, 9, 10, 11, 12, and 13). A t-test was performed on the null hypothesis, which stated that the mulberry leaf extract’s concentration did not affect the information entropy of the transcriptomes of the ‘Sawa-J’ fat bodies, using R and lm (Samples 16, 17, 18, 19, 20, 21, 22, and 23). A t-test was performed on the null hypothesis, which stated that the hours after dissection did not affect the information entropy of the transcriptomes of the ‘Sawa-J’ fat bodies, using R and lm (Samples 31, 32, 33, 34, 35, 36, 37, 38, 39, 40, 41, 42, 43, and 44). Data were visualized using R.

## 3. Results

**Figure 1:**
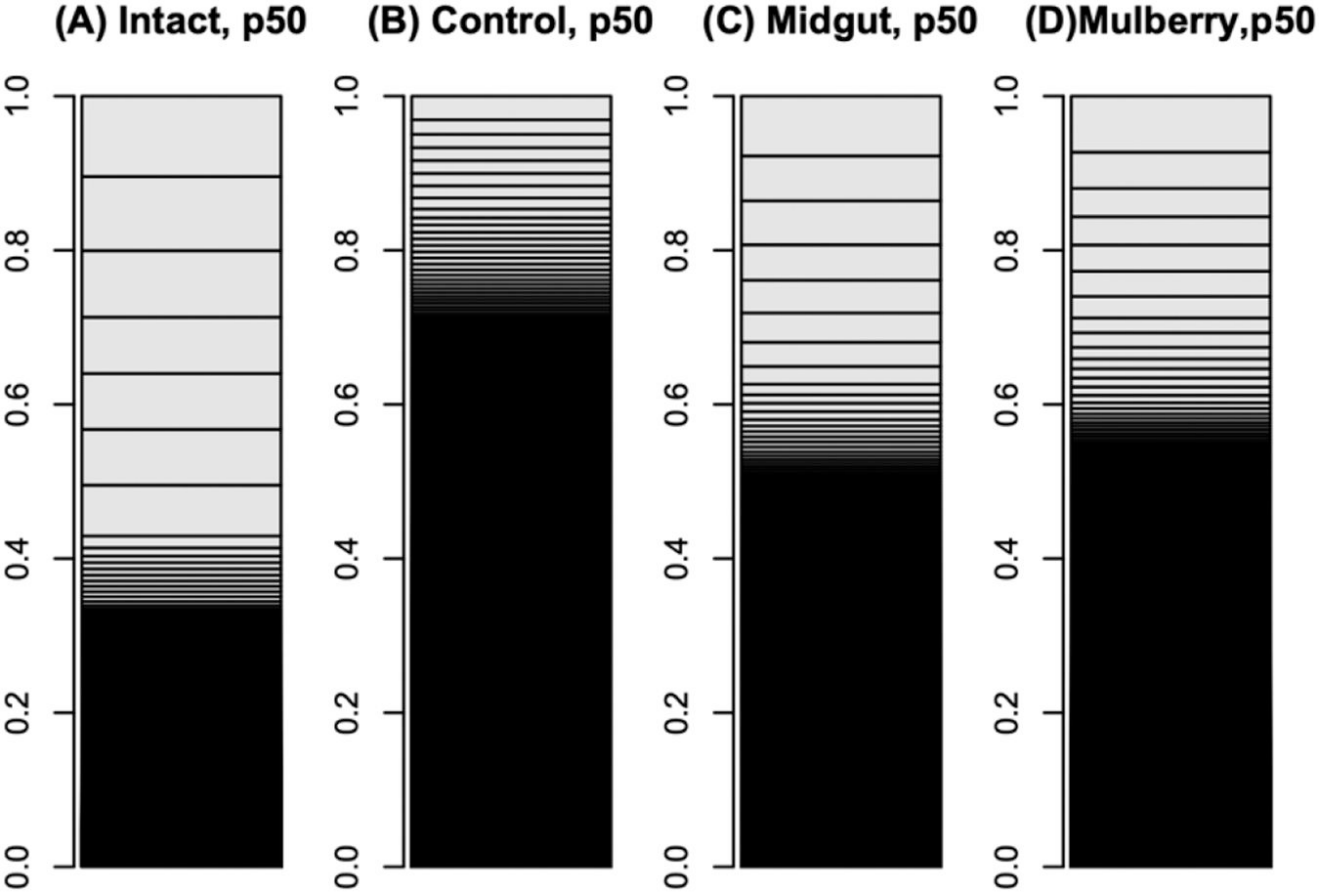
Bar charts of ‘p50’ silkworm fat-body transcriptomes. The occupation rates of genes in transcriptomes were expressed as bar charts. Heights of boxes in the bar charts indicate the occupation rates of the genes in the transcriptomes. Although more than 14,000 genes are included in these bar charts, most are invisible and are included in the black regions. (A) Transcriptome of intact silkworm fat-body tissue. (B) Transcriptome of fat-body tissues cultured for 90 h in MGM-450 insect medium. (C, D) Transcriptomes of fat-body tissues cultured for 80 h in MGM-450 insect medium and then an additional 10 h in medium supplemented with silkworm midgut (C) or mulberry leaf (D) extract.

**Figure 2:**
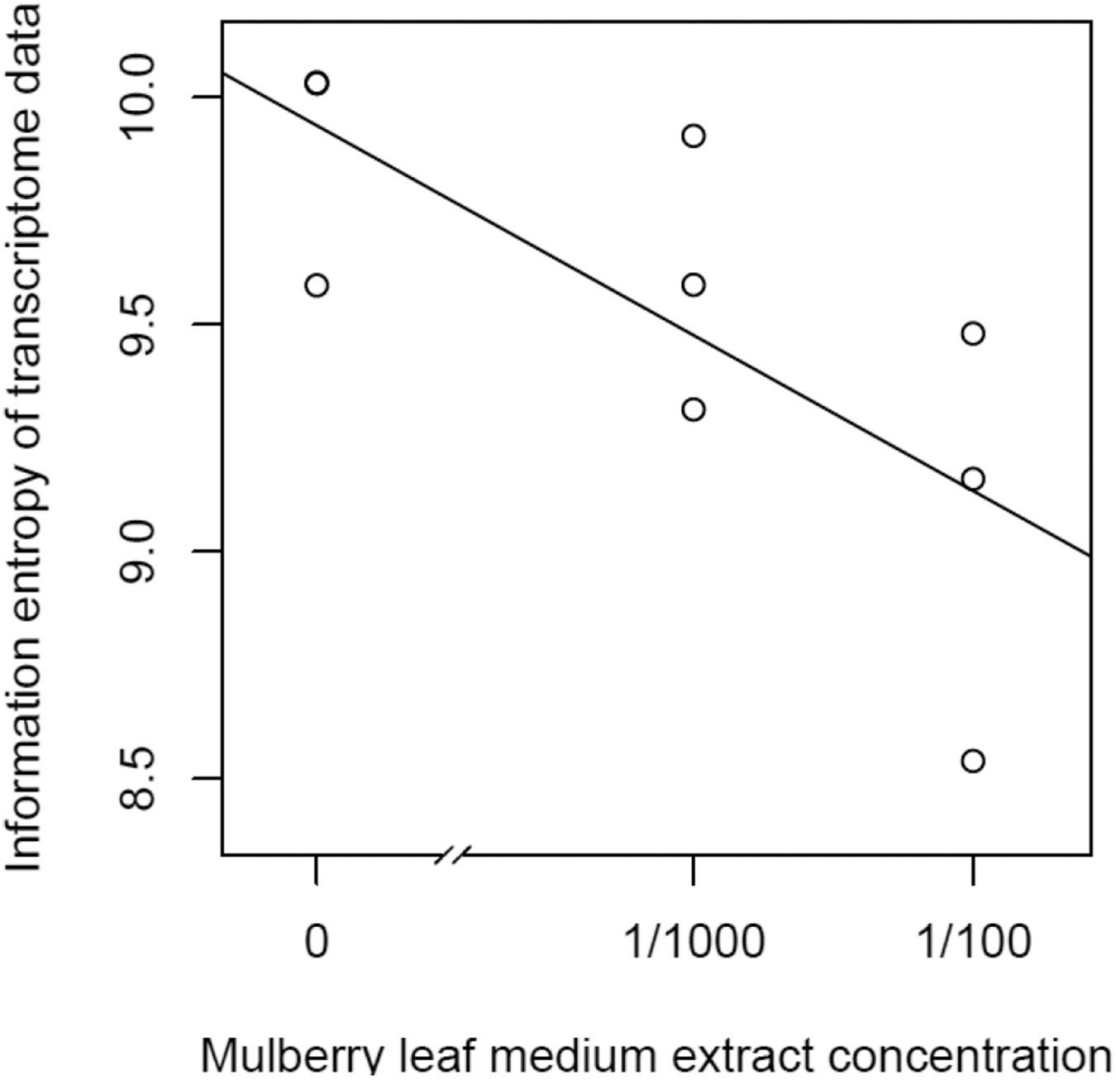
Scatter plot of the Mulberry leaf extract concentration *vs* the information entropy of the transcriptome data. Transcriptomes of ‘p50’ silkworm fat-body tissues that were cultured for 80 h in MGM-450 insect medium followed by 10 h in MGM-450 insect medium supplemented with Mulberry leaf extract.

**Figure 3:**
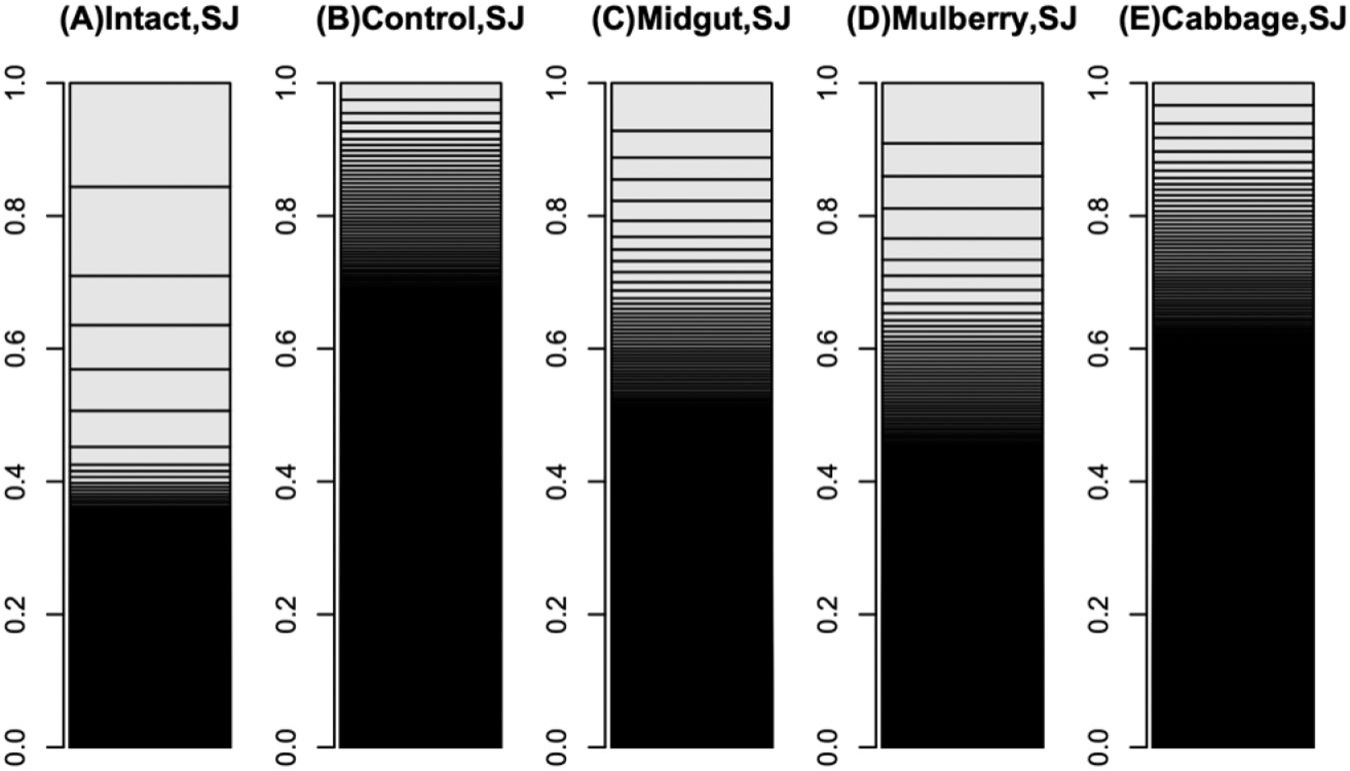
Bar charts of ‘Sawa-J’ silkworm fat-body transcriptomes. The occupation rates of genes in the transcriptomes were expressed as bar charts. Heights of boxes in the bar charts indicate the occupation rates of the genes in the transcriptomes. Although more than 14,000 genes are included in these bar charts, most are invisible and are included in the black regions. (A) Transcriptome of intact silkworm fat-body tissue. (B) Transcriptome of fat-body tissues cultured for 90 h in MGM-450 insect medium. (C–E) Transcriptomes of fat-body tissues cultured for 80 h in MGM-450 insect medium and then an additional 10 h in medium supplemented with silkworm midgut (C), mulberry leaf (D), or cabbage leaf (E) extract.

**Figure 4:**
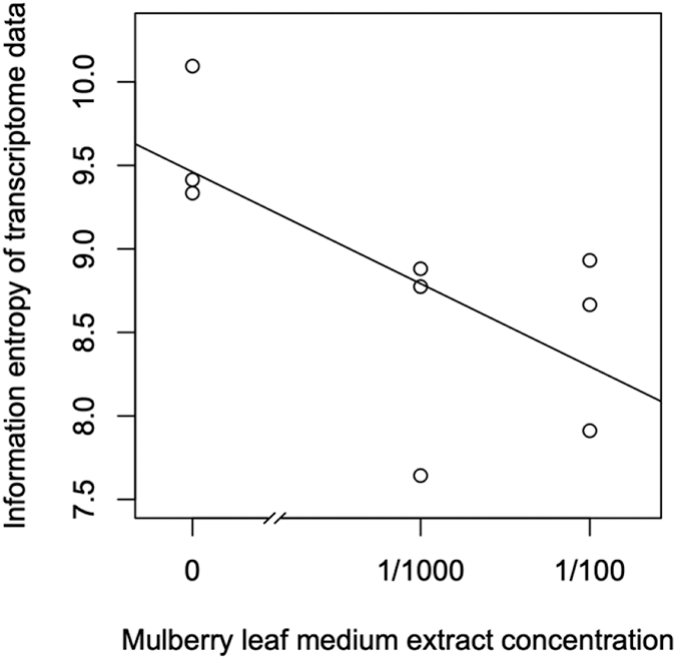
Scatter plot of Mulberry leaf extract concentrations *vs* the information entropy of the transcriptome data. Transcriptomes of ‘Sawa-J’ silkworm fat-body tissues that were cultured for 80 h in MGM-450 insect medium followed by 10 h in MGM-450 insect medium supplemented with Mulberry leaf extract.

**Figure 5:**
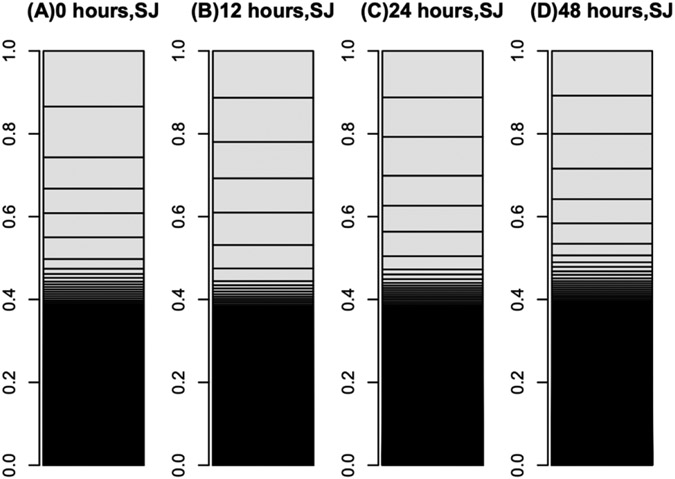
Bar charts of ‘Sawa-J’ silkworm fat-body transcriptomes. The occupation rates of genes in the transcriptomes were expressed as bar charts. Heights of boxes in the bar charts indicate the occupation rates of the genes in the transcriptomes. Although more than 14,000 genes are included in these bar charts, most are invisible and are included in the black regions. (A–D) Transcriptomes of fat-body tissues cultured for 0 (A), 12 (B), 24 (C), and 48 (D) h in MGM-450 insect medium.

**Figure 6:**
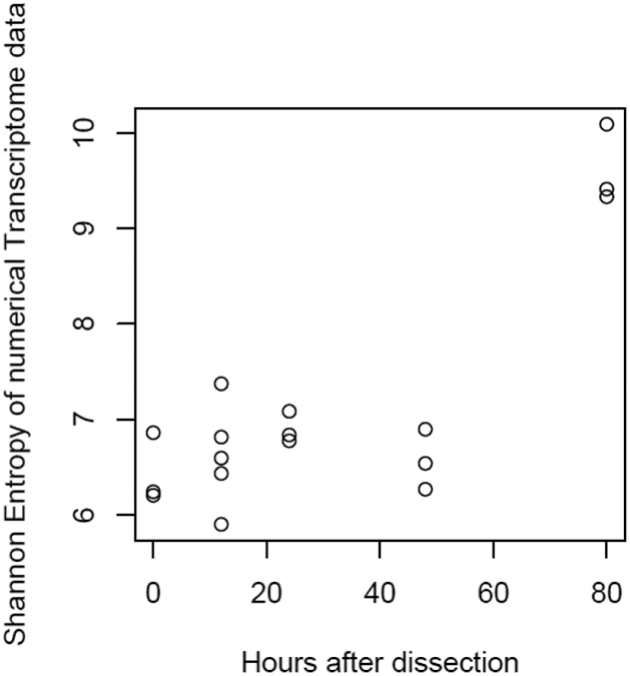
Scatter plot of culture time *vs* the information entropy of the transcriptome data. Transcriptomes of ‘Sawa-J’ silkworm fat-body tissues that were cultured for 0, 12, 24, 48 and 80 hours in MGM-450 insect medium.

The mulberry leaf extract decreased the information entropies of the transcriptomes of the ‘p50’ fat bodies (p = 0.025) (Figures 1A-D and 2). The silkworm midgut extract decreased the transcriptome diversity levels in the ‘p50’ fat bodies (Figure 1). The decrease in the information entropies of the transcriptomes of the ‘p50’ fat bodies cultured in the mulberry leaf extract were reproduced in another strain, ‘Sawa-J’ (p = 0.0457) (Figures 3A-E and 4). The silkworm midgut extract also decreased the transcriptome diversity levels in the ‘Sawa-J’ fat bodies (Figure 3). Fat-body dedifferentiation was not observed in the primary cultures until 48 h (Figures 5A-D and 6).

## 4. Discussion

The medium containing the midgut extract showed a bioactivity similar to that of the medium containing the mulberry leaf extract (Figures 1 and 3). This suggested that the bioactivity found in the midgut extract is derived from mulberry leaves. Thus, the midgut in living silkworms may release compounds from the mulberry leaves. Therefore, we hypothesized that components of mulberry leaves affect the fat bodies in the living organisms. The information entropies of the transcriptomes of the fat bodies decreased when primary culture-dependent dedifferentiated fat bodies were cultured in the mulberry leaf extract (Figures 2 and 4). The mulberry leaf extract-dependent tissue re-differentiation was shown in two silkworm strains (‘p50’ and ‘Sawa-J’). Thus, the previously discovered tissue responses to phenobarbital and permethrin may not have been caused by compound-specific bioactivity but by stimulating the *in vivo* control system, which originally functioned using components of mulberry leaves. This indicates that monophagous animals use components of their special feed not only as nutrients but, like hormones, to control homeostasis. Because maintaining a system that ensures a constant output, regardless of the input, requires a cost, there may be situations where a system that performs only the fixed processing of the input is selected. As a result of choosing the latter, monophasic animals may have lost the system that allows any food to be used in growth and development. In fact, silkworms grow poorly when raised on artificial diets that do not contain mulberry. In other insects, dietary changes result in changed outputs, such as pheromone secretions. One lepidopteran insect changes its secreted sex pheromone components in response to being fed artificial diets [22]. ‘Sawa-J’ is an abnormal feeding-habit strain that feeds on an artificial diet lacking mulberry leaf components [23,24]. It was expected that the fat bodies from members of this strain would differentiate in response to components derived from plants other than mulberry, but the cabbage extract had no effect (Figure 3). Micro-particles of a uniform shape were observed in ‘Sawa-J’ tissue cultures. We revealed that the cultures were infected with NPV, and we identified many NPV sequences (GenBank: JQ071499.1) using RNA-seq. The NPV infection did not interrupt the mulberry leaf-dependent re-differentiation of the fat bodies. In this experiment, mulberry leaf extract was used in the medium to develop a culture environment, and thus, fat-body transcriptomes, that was closer to the *in vivo* environment. However, the information entropy of the *in vivo* fat-body transcriptome is clearly less than that of the transcriptome of a fat body cultured in a medium supplemented with the mulberry leaf extract. Thus, the *in vivo*-like culture techniques for silkworm fat bodies can be improved. Here, we revealed that cultured fat bodies did not immediately dedifferentiate (< 48 h). This contrasts the cell re-differentiation seen within 10 h when cells were transferred from a medium supplemented with Phenobarbital and permethrin to a drug-deficient medium [14]. By clarifying this difference, we hope to obtain information that will allow us to mimic the *in vivo* environment in an *in vitro* culture system. In this decade, several studies [15,25-27] following our research [14] repeatedly measured the degree of cellular dedifferentiation and differentiation as a transcriptome Shannon entropy. The Shannon entropy is a kind of alpha diversity in ecology [28], and the transcriptome Shannon entropy is simply transcriptome diversity [14,29]. It is not incorrect to call it the alpha diversity of the transcriptome, but that would leave its biological significance undefined, as would each principal component that came up in the principal component analysis. Since we can quantitatively assess, judge, and define that dedifferentiation is an increase in the Shannon entropy of the transcriptome and differentiation is a decrease in the Shannon entropy of the transcriptome, it is more accurate to position the value not as a mere bioinformatics measure; however, as a number with obvious biological and bioengineering significance, such as viable cell rate, cell density, specific growth rate, or pcd (pg/cell/day). Here we call the quantitative value of cellular dedifferentiation and differentiation “liberality,” since a previous study explained the changes were happening to cultured cells as “libère” [30-33].

## 5. Conclusions

To investigate the possibility that feed-derived components are essential to the normal lives of monophagous silkworm, mulberry leaf components were added to the dedifferentiating primary cultures of silkworm fat bodies. Using mulberry leaf extract, we were able to differentiate silkworm fat bodies in primary cultures.

## Supplementary Material

1
