# Supplementary material for: In vivo-like Culture of Monophagous Animal Organ using Dietary Components
Source: J Biotechnol Biomed. Author manuscript; Available in PMC 2023 Mar 3. (PMC9983661; doi:10.26502/jbb.2642-91280070)
Supplement: 1 [file NIHMS1874003-supplement-1.pdf]

Sample ID	Shannon Entropy	Culture Condition	Strain
1	6.865143	Not cultured	p50
2	5.914764	Not cultured	p50
3	6.605657	Not cultured	p50
4	6.454235	Not cultured	p50
5	10.03083	Control	p50
6	10.03309	Control	p50
7	9.586332	Control	p50
8	9.587584	MulberryLeaf1/1000	p50
9	9.915635	MulberryLeaf1/1000	p50
10	9.313175	MulberryLeaf1/1000	p50
11	9.1602	MulberryLeaf1/100	p50
12	9.480333	MulberryLeaf1/100	p50
13	8.539101	MulberryLeaf1/100	p50
14	8.255311	Midgut	p50
15	6.532235	Not cultured	Sawa-J
16	10.09469	Control	Sawa-J
17	9.414184	Control	Sawa-J
18	9.33448	Control	Sawa-J
19	8.774243	MulberryLeaf1/1000	Sawa-J
20	8.881261	MulberryLeaf1/1000	Sawa-J
21	7.642716	MulberryLeaf1/1000	Sawa-J
22	8.665842	MulberryLeaf1/100	Sawa-J
23	8.931488	MulberryLeaf1/100	Sawa-J
24	7.911349	MulberryLeaf1/100	Sawa-J

25	8.412092	Midgut	Sawa-J
26	8.295697	Midgut	Sawa-J
27	8.95734	Midgut	Sawa-J
28	9.434283	CabbageLeaf	Sawa-J
29	9.661787	CabbageLeaf	Sawa-J
30	9.491179	CabbageLeaf	Sawa-J
31	6.859353	Not cultured	Sawa-J
32	6.202299	Not cultured	Sawa-J
33	6.239494	Not cultured	Sawa-J
34	5.89826	Culture12hours	Sawa-J
35	6.813056	Culture12hours	Sawa-J
36	6.593159	Culture12hours	Sawa-J
37	6.432197	Culture12hours	Sawa-J
38	7.37291	Culture12hours	Sawa-J
39	6.774276	Culture24hours	Sawa-J
40	7.083945	Culture24hours	Sawa-J
41	6.835882	Culture24hours	Sawa-J
42	6.537758	Culture48hours	Sawa-J
43	6.895498	Culture48hours	Sawa-J
44	6.266251	Culture48hours	Sawa-J

Supplementary Material 1: Information entropy of transcriptomes. In total, 44 transcriptomes were sequenced. Sample IDs, information entropies, culture conditions, and strains are summarized.
